# Acquisition pattern and the role of vocabulary and language experience in the acquisition of inflectional grammar by Mandarin-English speaking preschoolers

**DOI:** 10.3389/fpsyg.2024.1302044

**Published:** 2024-02-15

**Authors:** Nan Xu Rattanasone, Jae-Hyun Kim

**Affiliations:** ^1^Centre for Language Sciences, Faculty of Medicine, Health and Human Sciences, Macquarie University, Sydney, NSW, Australia; ^2^Multilingualism Research Centre, Faculty of Medicine, Health and Human Sciences, Macquarie University, Sydney, NSW, Australia; ^3^Health and Human Sciences, Department of Linguistics, Faculty of Medicine, Macquarie University, Sydney, NSW, Australia

**Keywords:** bilingual acquisition, inflectional grammar, Mandarin, English, preschoolers, language experience

## Abstract

Australian Mandarin-English bilingual preschoolers must acquire linguistic structures that occur only in the community language (e.g., English inflectional grammar). This study investigated how they acquire such structures and any relationship between linguistic knowledge and language experience on their performance. Twenty 4–6-year-olds showed known monolingual acquisition patterns with good performance for producing the progressive, developing ability for plurals, but only emerging ability for past and present tense. Better performance was related to a larger English vocabulary, more mixed language input and use, but less Mandarin input and use. On average, these children received less than 50% input in English and were performing behind monolinguals.

## 1 Introduction

In Australia, many bilingual children begin to learn English (the community language) as an additional language at childcare/preschool. In New South Wales, where Sydney is located, 37.7% of children speak a language other than English (NSW Department of Education, 2022). Many children speak Mandarin as a heritage language as it is Australia’s most spoken heritage language (ABS, 2021). However, Mandarin and English are typologically distant languages, with many structures found only in English. Whether acquiring these new structures is the same, delayed or different from monolingual patterns and the role of bilingual language input and use on acquisition is currently unclear. This study examined a cohort of Mandarin-English speaking 4-6-year-olds, similar in age of exposure to English and parental education, to investigate the acquisition pattern for English inflectional grammar and any relationship with language input and use.

### 1.1 Acquisition of inflectional grammar

Mandarin and English differ across phonological and morphological structures. While Mandarin words have predominantly simple syllable structures (CV), e.g., *ma*, English words typically end in a consonant [CVC(C)], e.g., *cat*, but could also end in consonant clusters that carry inflections, e.g., /test/ in cats. Grammatical information in Mandarin is expressed in a noun phrase or compound, e.g., three cats as 三只猫 (number + classifier + noun) or kicked as 踢了 (verb + perfective tense particle). The word-final position in English therefore has a high functional load, but this is not so for Mandarin where codas are typically absent (except two nasals). Therefore, both the simple syllabic structure of words and the absence of inflections in Mandarin could act to constrain the learning of English inflections, a new linguistic structure occurring in a non-salient word position. Indeed, Mandarin-speaking school-aged children learning English inflectional grammar are behind their monolingual peers after 5 to 6 years of English immersion (Jia, 2003; Jia and Fuse, 2007; Paradis et al., 2016).

English-speaking monolingual children, on the other hand, acquire English grammatical inflections shortly after producing their first words, with 2-year-olds producing plurals in obligatory contexts, e.g., *some cats* (Brown, 1973). There is also an order of acquisition for the plural allomorphs with the more frequently occurring segmentals, i.e., –*s* in *cats*, acquired earlier than the less frequently occurring syllabic plural allomorph, i.e., –*es* in *horses* (Davies et al., 2017, 2019, 2020). Verbal inflections also emerge around the same time, with the progressive (–*ing*) emerging first at around 2 years, followed by the past and present tense morphemes at around 5 years (Berko, 1958; Brown, 1973; de Villiers and de Villiers, 1973).

Studies with bilingual preschoolers speaking a mix of different home languages have shown quantitatively delayed but qualitatively monolingual-like patterns of acquiring inflectional grammar (Hammer et al., 2014). However, studies examining the pattern of plural acquisition in Australian bilingual preschoolers have shown different patterns for those speaking a heritage language *with* and *without* inflections. While those speaking a heritage language *with* inflections are on their way to acquiring plural grammar but behind monolingual performance, preschoolers speaking *isolating* languages (e.g., Chinese Mandarin, Cantonese, etc.) are just beginning to show an emerging understanding of the more frequently occurring segmental plural form (Xu Rattanasone et al., 2016, 2024). Similar effects have been reported for present and past tense (Paradis, 2011; Blom et al., 2012). This raises questions about whether bilingual children acquiring structures in only the community language might show delayed or different patterns to monolinguals.

### 1.2 Language input and bilingual development

Bilingual children’s language experiences differ fundamentally from monolingual experiences. Mixing languages is a common practice in bilingual families. Mixing can occur within a single or across different utterances, across topics and contexts, or among different speakers. In large bilingual societies such as Vancouver, Canada, up to 90% of language input to young children can be mixed (Byers-Heinlein, 2013), but this could vary considerably across societies and families (Place and Hoff, 2011, 2016; Bail et al., 2015; Kremin et al., 2022). However, despite the ubiquitous nature of mixed language input, few studies have examined the relationship between language mixing and language development in bilingual children, and the small number of existing studies show mixed findings.

Studies that use *parental reports* (questionnaires or diaries) show either no relationship or a negative relationship between mixed language input and early language development. Among Spanish-English speaking preschoolers, mixed language input (as a percentage over total input including English and Spanish only in daily 30-min blocks) had no relationship with bilingual vocabulary or grammar (Place and Hoff, 2011, 2016). However, among diverse heritage language speakers, increased mixed language input was associated with reduced English vocabulary (Byers-Heinlein, 2013). A study using measures derived from *recorded samples* of Spanish-English speaking parent and child interactions showed positive relationships between language development and mixed language input, but only within (not across) sentences (Bail et al., 2015). The authors reported that mixing within a sentence involved repeating the same word in both languages, providing translation equivalents. While this might facilitate vocabulary development, such opportunities for language transfer do not exist for linguistic structures that occur only in one language, e.g., inflectional grammar.

Other studies have examined dual language input separately. For example, Welsh has one of the most complex inflectional systems, and by 11 years, bilingual children from Welsh-speaking homes were approaching adult performance in producing plurals, but those from English-speaking homes were producing less than 50% on all plural inflections (Thomas et al., 2014). On the other hand, French-English bilingual preschoolers from Montreal who received at least 50% input in English performed like their English-speaking monolingual peers on morphosyntax (Thordardottir, 2015; also see Thordardottir, 2011, for similar effects on vocabulary). For very young children, the amount of language exposure to an additional language “trumps” age of acquisition effects (Thordardottir, 2019). This suggests that transferring inflectional knowledge from the more complex French system to a simpler English system might have resulted in earlier acquisition. However, with no inflectional system to transfer, such benefits are not available to Mandarin-speaking children acquiring English. These studies raise questions about the language acquisition pattern and role of input for Mandarin-English speaking children.

### 1.3 Vocabulary and inflectional grammar

All studies so far have used vocabulary as an early measure of linguistic skills. In monolingual children, having a larger vocabulary is typically linked to more advanced language development, already evident in language processing skills within the first 2 years of life (Fernald et al., 2013), with long-lasting implications for later academic performance (Hoff, 2013). In L2 English learning school-aged children, those with a larger vocabulary also performed better in producing inflectional grammar, e.g., third-person singular –*s* (Blom et al., 2012), suggesting that vocabulary size also plays a key role in acquiring inflectional grammar.

A larger vocabulary may provide greater access to morphologically complex word forms, including various allomorphs and their stem forms (i.e., *cat*-*cats* and *horse*-*horses*). Understanding internal word structure and the grammatical function of morphemes might be essential for acquiring inflectional grammar. However, for Mandarin-English bilinguals, English vocabulary is their only access to inflections, and having an extensive English vocabulary might be advantageous for acquiring inflectional grammar.

### 1.4 This study

This study examined the acquisition of English inflectional morphemes and related linguistic and language factors in Mandarin-English speaking bilingual children in Sydney, Australia. These children typically enter childcare/preschool with little or no English, begin to learn inflectional grammar for the first time, and will continue learning English in the community. In areas like Sydney, with large Mandarin-speaking communities, native Mandarin speakers are often embedded in childcare/education settings as early childhood educators. These circumstances provide a relatively homogenous population of children to probe whether Mandarin-English bilingual children show quantitative or qualitative differences from monolingual language acquisition. We systematically measured their acquisition of inflectional morphemes, including the segmental and syllabic forms and interpreted their performance against known monolingual patterns. Additionally, we explored the relationship of language input and use (Mixed, English and Mandarin) with performance on inflectional morphology. Parents provided diary reports of hourly languages heard and used by the child during the day in English and Mandarin only, and Mixed language input if both languages were used during the activity/h.

This study will address the following two research questions (RQ): First, do Mandarin-English bilingual children show monolingual patterns of acquisition for inflectional grammar? Second, are vocabulary and daily language input and use (Mixed, English, Mandarin) associated with performance in English inflectional morphemes? The following hypotheses are developed to test these RQs:

Hypothesis 1a: If Mandarin-English bilingual children perform like their monolingual peers, they should show the best performance on progressive, followed by the plurals, then the present and past tense morphemes.

Hypothesis 1b: They should also show better performance on the segmental allomorphs (e.g., *cats*) than syllabic (e.g., *horses*) forms.

Hypothesis 2: English vocabulary is expected to show a positive relationship with performance in English inflectional morphemes.

Given the inconsistent past findings, however, any possible relationship between daily language input and use (Mixed, English, Mandarin) and performance in English inflectional morphemes will be explored.

## 2 Methods

### 2.1 Participants

Twenty 4- to 6-year-olds (Mean = 4;11, Range: 4;2–6;4; 13 girls) speaking Mandarin at home and learning English in preschool/school participated in the study.^1^ All children sampled were born in Australia except for one child who arrived as a 10-month-old. The mean age of acquisition for English was 19 months (Range: 8–35), as indicated by the age of enrolment into an English-speaking childcare center. All primary caregivers (18 mothers and two fathers) were native speakers of Mandarin from Mainland China. Two parents received vocational training, eight completed an undergraduate degree and 10 postgraduate degrees. Of these, 14 received their highest level of training in Australia and six in China (two in English and four in Mandarin). This project has been approved by the Macquarie University Human Research Ethics Committee (approval number 52020662715782).

### 2.2 Materials

All parents completed a demographic and language history questionnaire. Parents also provided a seven-day diary of their children’s hourly activities, including any interlocutors and languages heard and used (Both/Mixed, English, or Mandarin).^2^

Expressive vocabulary in both English and Mandarin was elicited using two lists, one for each language, with each list containing 50 words (half nouns and half verbs; Jia et al., 2014). The task has been used with similar-aged Mandarin-English bilingual children (5–7-year-olds) and showed sensitivity to language input effects (Jia et al., 2014),^3^ but it is not a standardized clinical tool.

A task for eliciting English grammatical inflections was designed containing the progressive, plural, past, and present tense morphemes with 10 items per morpheme type. Half were segmental (e.g., *cats*) and half syllabic (e.g., *horses*) for plural, past, and present tense. This task was intended to measure children’s productive use of English inflections (Ren et al., 2018; Xu Rattanasone et al., 2024) and is not a standardized clinical tool to indicate atypical/disordered acquisition. Children were provided with syntactic and semantic contexts that require the inflected forms of nouns or verbs.^4^ A female native Australian English speaker recorded all audio recordings played to children.

### 2.3 Procedure

All children were assessed online using Zoom for expressive vocabulary in each language and the inflectional morphemes task in English. Two native speakers of Mandarin administered all tasks and had the children and parents in view during the session. All parents were explicitly instructed not to provide answers during the session. In the expressive vocabulary task, children were shown pictures and asked to name them one at a time. If the child produced the wrong item, they were asked if the item had another name, and if the child refused to produce a label after three prompts, the researcher moved on to the next item. The task was completed in Mandarin first, followed by English. The order of presentation was not counterbalanced as the study was conducted remotely online, in the children’s homes, with help from their Mandarin-speaking parents, so all sessions began in Mandarin. Also, having their parents encourage them to participate in the home language helped ensure better engagement with the task. The English grammatical inflections task was given after the expressive vocabulary task. At the beginning of each block, two practice trials were given where children who could not produce the response were played pre-recorded target responses and asked to repeat them in full before progressing to the test trials. No feedback was provided during the test trials. Each type of morpheme was blocked and presented in a fixed order: (1) progressive, (2) present, (3) plurals, and (4) past tense.

## 3 Results

To address Hypothesis 1a (If Mandarin-English bilingual children perform like their monolingual peers, they should show the best performance on progressive, followed by the plurals, then the present and past tense morphemes), two General Linear Mixed-Effects Models were carried out in JAMOVI (The Jamovi Project, 2021) using R (R Core Team, 2021) and the GAMLj jamovi module (Gallucci, 2019). Age of acquisition was included as a covariate. Models with random intercepts for participants were fitted as these were the only models that converged. Restricted maximum likelihood was used to fit the model, and degrees of freedom were adjusted with the Satterthwaite method. Normal distribution of model residuals was observed.

For the first model, performance on the four types of morphemes was fitted with polynomials (linear, quadratic, and cubic) with the morphemes dummy coded as 1 = Progressive, 2 = Plural, 3 = Present, and 4 = Past tense. The results detected only a significant negative linear trend (see Table 1A), suggesting children’s performance decreased from Progressive to Past tense. *Post hoc* analysis with Bonferroni adjustment to *p*-values (see Table 1B for results) showed that while the Progressive (M = 80.8%) did not differ significantly from the Plural (M = 62.0%), it was produced significantly more than both Present (M = 45.3%) and Past tense (M = 31.5%). While performance on the Plural did not differ significantly from Present tense, it was significantly higher than Past tense. These results suggest that children performed better in the Progressive and Plural than in Present and Past tense.

**Table 1:** **(A)** Fixed effects parameter estimates for the percentage of morphemes produced by children with significant results in bold; **(B)** *Post hoc* comparisons for the percentage of morphemes produced by children with significant results in bold.

(A)								
			95% confidence interval					
Effect	Estimate	SE	Lower	Upper	df	*t*	*p*	
(Intercept)	54.875	7.351	40.470	69.280	18.400	7.465	< 0.001	
AoA	0.444	0.897	−1.310	2.200	18.000	0.495	0.627	
Linear	**−36.783**	**5.754**	**−48.060**	**−25.510**	**117.000**	**−6.393**	**< 0.001**	***
quadratic	2.500	5.342	−7.970	12.970	117.000	0.468	0.641	
cubic	0.224	4.896	−9.370	9.820	117.000	0.046	0.964	

(B)								
Comparison								
Morpheme		Morpheme	Difference	SE	*t*	df	*p_bonferroni_*	
Progressive	-	Plural	18.700	8.280	2.270	117	0.152	
Progressive	**-**	**Present**	**35.500**	**8.280**	**4.290**	**117**	**< 0.001**	***
Progressive	**-**	**Past**	**49.200**	**8.280**	**5.950**	**117**	**< 0.001**	***
Plural	-	Present	16.700	6.760	2.480	117	0.088	
Plural	**-**	**Past**	**30.500**	**6.760**	**4.510**	**117**	**< 0.001**	***
Present	-	Past	13.700	6.760	2.030	117	0.265	

*R*-code: Score~1 + AoA + Morpheme_Code + (1 | Participant); AoA, age of acquisition; In bold are significant findings, ^***^p < 0.001.

A second model tested Hypothesis 1b (If Mandarin-English bilingual children perform like their monolingual peers, showing better performance on the segmental allomorphs (e.g., *cats*) than syllabic (e.g., *horses*) forms), the three morpheme types (Plural, Present and Past tense) and allomorphs were entered as a fixed factor. The results in Table 2 show a significant linear trend and a significant main effect of allomorph type and no interactions. The results were consistent with the first model but further suggested that, in addition to the linear decrease in performance from Plural to Past tense, children also produced segmental forms (M = 56.5%) more accurately than syllabic forms (M = 36.0%).

**Table 2:** Fixed effects parameter estimates for the percentage of allomorphs produced by children with significant results in bold.

			95% confidence interval					
Effect	Estimate	SE	Lower	Upper	df	*t*	*p*	
(Intercept)	46.250	7.856	30.850	61.650	18	5.888	< 0.001	
AoA	0.410	0.964	−1.480	2.300	18	0.425	0.676	
Allomorph (syllabic − segmental)	**−20.500**	**5.244**	**−30.780**	**−10.220**	**95**	**−3.909**	**< 0.001**	***
Linear	**−21.567**	**4.541**	**−30.470**	**−12.670**	**95**	**−4.749**	**< 0.001**	***
Quadratic	1.225	4.541	−7.680	10.130	95	0.270	0.788	
Allomorph (syllabic − segmental) ✻ linear	0.707	9.082	−17.090	18.510	95	0.078	0.938	
Allomorph (syllabic − segmental) ✻ quadratic	7.348	9.082	−10.450	25.150	95	0.809	0.420	

R-code: Score~1 + AoA + Allomorph + Morpheme_Code + Allomorph:Morpheme_Code + (1 | Participant); AoA, age of acquisition; In bold are significant findings, ^***^p < 0.001.

To address Hypothesis 2: English vocabulary is expected to show a positive relationship with performance in English inflectional morphemes. Any possible relationships between daily language input and use (Mixed, English, Mandarin) and performance in English inflectional morphemes will also be explored. Three analyses will be presented: (1) Performance on vocabulary, (2) language input and use, and (3) the correlations.

*Vocabulary:* Vocabulary scores were analyzed using a General Linear Model with Language (Mandarin, English) entered as the fixed factor (see Table 3 for results). The percentage of words correctly produced in each language was calculated. With alpha set at 0.05, the analysis detected a significant main effect of language, with children producing more English than Mandarin words.

**Table 3:** Fixed effects parameter estimates for children’s percentage of vocabulary score in English compared to Mandarin.

		95% confidence interval						
Effect	Estimate	SE	Lower	Upper	β	df	*t*	*p*
(Intercept)	81.950	1.230	79.500	84.440	0.000	3866.680	< 0.001	
**Mandarin—English**	**−7.630**	**2.460**	**−12.600**	**−2.660**	**−0.889**	**38**	**−3.110**	0.004**

^***^p < 0.001.

With significant results in bold.

*Language Input and Use:* A General Linear Model was conducted on the language diary data with means derived for Input vs. Use (Mixed, English, Mandarin as proportion of total input) as fixed factors and alpha set at 0.05 (see Table 4A for results). The analysis (see Table 4B for results) detected only a significant main effect of language with linear and quadratic trends. Post-hoc analysis with Bonferroni adjustment to *p*-values shows that English input and use (M = 46.952%) is significantly greater than both Mixed (M = 26.326%) and Mandarin (M = 26.720%) input and use, but the latter two did not differ significantly.

**Table 4:** **(A)** Fixed effects parameter estimates for the proportion of mixed, English, and Mandarin input and use with significant effects in bold; **(B)** *Post hoc* analysis for language mixed, English, and Mandarin input and use with significant effects in bold.

(A)										
			95% Confidence Interval							
Effect	Estimate	SE	Lower	Upper	β	df		*t*	*p*	
(Intercept)	0.333	0.021	0.292	0.374	0.000	114	114	16.136	< 0.001	
Input − Use	0.000	0.029	−0.058	0.058	0.000	114	114	0.002	0.999	
Language linear	**−0.146**	**0.036**	**−0.217**	**−0.075**	**−0.602**	**114**	**114**	**−4.073**	**< 0.001**	***
Language quadratic	**0.081**	**0.036**	**0.010**	**0.152**	**0.335**	**114**	**114**	**2.265**	**0.025**	*
Input − Use ✻ linear	0.013	0.051	−0.087	0.113	0.054	114	114	0.260	0.795	
Input − Use ✻ quadratic	0.028	0.051	−0.072	0.129	0.117	114	114	0.561	0.576	

(B)								
Comparison								
Language		Language	Difference	SE	*t*	df	*p_bonferroni_*	
English	**-**	Mandarin	0.202	0.051	3.998	**114**	**< 0.001**	***
English	**-**	Mixed	0.206	0.051	4.073	**114**	**< 0.001**	***
Mandarin	-	Mixed	0.004	0.051	0.075	114	1.000	

^*^p ≤ 0.05, ^***^p ≤ 0.01.

Pearson’s bivariate correlation analysis was conducted using percentage correct scores on the inflectional morphemes task, English and Mandarin vocabulary, and proportion of Mixed, English, and Mandarin language input and use (see Table 5 for results; age of acquisition was not included given that it was not significant in the two earlier models).

**Table 5:** Correlations of children’s percentage correct scores on Inflectional Grammar and English and Mandarin Vocabulary, as well as Mixed, English, and Mandarin language Input and Use scores derived from diary reports with significant results in bold.

	Inflection		Eng Vocab		Mand Vocab		Input Mixed		Input Eng		Input Mand		Use Mixed		Use Eng	Use Mand
Inflection	—															
Eng Vocab	**0.784**	***	—													
Mand Vocab	0.097		0.022		—											
Input Mixed	**0.725**	***	0.383		0.140		—									
Input Eng	0.036		0.359		−0.281		−0.461		—							
Input Mand	**−0.800**	***	**−0.687**	**	0.063		**−0.724**	**	−0.276		—					
Use Mixed	**0.562**	*	0.296		0.071		**0.888**	***	−0.441		**−0.622**	**	—			
Use Eng	0.089		0.317		−0.298		−0.349		**0.959**	***	−0.365		−0.317		—	
Use Mand	**−0.630**	**	**−0.517**	*	0.033		**−0.638**	**	−0.349		**0.924**	***	**−0.763**	***	−0.478	—

^*^p < 0.05, ^**^p < 0.01, ^***^p < 0.001.

First, correlations among language input measures showed a significant *negative* relationship between Mixed language input and Mandarin input (moderate relationship). Correlations among the language use measures showed a significant *negative* relationship between Mixed language use and Mandarin use (strong relationship). These results suggest that children who received more Mixed language received less Mandarin-only input, and those who used more Mixed languages also used less Mandarin. Across language input and use measures, significant *positive* relationships were detected for Mixed language input and use (strong relationship), English input and use (strong relationship) and Mandarin input and use (strong relationship). However, a significant *negative* relationship was detected between Mixed language input and Mandarin use (moderate relationship). These results suggest a tight relationship between the same types of language input and use, but those who received more Mixed language input used Mandarin less.

Second, the correlations between vocabulary and language input and use showed significant *negative* relationships between English vocabulary and Mandarin input and use (moderate relationships). Mandarin vocabulary did not have any significant correlations. These results suggest that children who received and used more Mandarin had a smaller English vocabulary.

Finally, correlations between children’s performance on inflectional morphemes with vocabulary and language input and use showed several significant *positive* correlations between children’s performance on inflectional morphemes and (1) their English vocabulary (strong relationship), (2) the amount of Mixed language input (moderate relationship) and (3) Mixed language use (moderate relationship). Significant *negative* correlations were detected between children’s performance on inflectional morphemes and Mandarin input and use (strong and moderate relationships). These results indicate that children who performed better on the inflectional morpheme task had larger English vocabulary and heard and used more Mixed language input. Additionally, those who heard and used more Mandarin performed worse in the inflectional grammar task.

## 4 Discussion

This study examined whether bilingual Mandarin-English speaking 4–6-year-olds demonstrate monolingual-like patterns when learning inflectional morphemes (missing in Mandarin) and any relationship with dual language vocabulary and input and use.

### 4.1 Mandarin-English bilingual children show monolingual patterns of acquisition

First, Mandarin-speaking preschoolers showed established monolingual patterns: (1) performing best on the progressive (80%), followed by the plural (62%), then present (45%) and past tense (31%); (2) performing better on the segmental than syllabic allomorphs (Davies et al., 2017, 2020). While these children are showing good progress in acquiring the progressive and plural, they are just showing emerging knowledge of the present and past tense. The results suggest that bilingual children acquire linguistic structures found only in English, the community language, like their monolingual peers, but the developmental course is protracted, i.e., later but not different (similar to Jia, 2003; Jia and Fuse, 2007; Paradis et al., 2016 in the US and Canada). However, it remains to be seen when these children will catch up to their monolingual English-speaking peers.

These results have important implications for both research and application. Unlike older sequential bilinguals learning L2 English at school, younger bilingual preschoolers can begin to acquire new linguistic structures over a brief period of exposure to English during childcare. Indeed, some research has suggested that children’s capacity to learn new linguistic structures in a second language diminishes during preschool (Meisel, 2018). However, English has a small inventory of grammatical inflections and may be easier to learn than other systems—which needs further investigation.

### 4.2 English vocabulary and language input and use

Second, children’s performance on English inflectional morphemes was linked to larger English vocabulary, supporting existing literature (e.g., Blom et al., 2012). However, no relationship was found between performance on English inflectional morphemes and Mandarin vocabulary. This suggests that exposure to morphologically complex inflected words is critical for acquiring inflectional grammar. Children speaking a heritage language without inflections may need additional early language support during preschool, e.g., explicit teaching of inflectional grammar.

Third, more Mixed language input is related to better performance, but more Mandarin input is related to poorer performance on English inflectional morphemes. Our findings add to the existing body of equivocal evidence on Mixed language input and language development (Place and Hoff, 2011, 2016; Byers-Heinlein, 2013; Bail et al., 2015) and are inconsistent with findings from a large heterogeneous sample of bilingual primary-aged children from diverse heritage languages, showing that English grammatical inflections are not sensitive to the effect of language input and use (De Cat, 2020). However, unlike these studies, the current study is the first to examine a relatively homogenous sample of children in chronological age and age of acquisition of a community language, speaking an isolating heritage language.

Our findings for Mandarin input are relatively straightforward but are more complex for Mixed and English language input. Given that Mandarin does not have inflectional morphemes and time is finite, more exposure to Mandarin would lead to less exposure to English and later acquisition. Mixed language use in our sample may be driven by the generally high level of parental education and mostly in English. Mixed language input could reflect higher English proficiency. Indeed, higher maternal education in English is linked to better English development in Spanish-English bilinguals (Hoff et al., 2018). We observed through the diary reports and recordings of the mother–child interactions (in preparation) that home-based dyadic interactions often involve explicit learning instructions involving Mixed language input. Parent-led home learning could be a cultural practice or reflect higher parental education. This project could not tease apart these issues and any differential effects of mixed language use during learning vs. play-based activities and language development. Future research should describe mixed language input and use in more detail, e.g., lexical and linguistic diversity, to better understand its role in bilingual language acquisition.

The lack of relationship between English input and performance on English inflectional morphemes initially appears counterintuitive. One explanation could be that the English input in our sample was low and/or not showing enough variation. Children received more Mandarin and Mixed than English input at home (27 and 24% of total input vs. 11% English). But despite receiving more English and little Mandarin or Mixed input in the community (35.6% English of total language input vs.1.8% and 1.2%), the combined English input is on average only 46.8% – below the 50% identified as sufficient to achieve monolingual performance (Thordardottir, 2015). While our sample of children received considerable mixed language input (25%), the proportion and quality of the English is unclear. Indeed, non-native input can impact children’s lexical and morphosyntactic development (Unsworth et al., 2019; Hoff et al., 2020). The *quality* and *quality* of mixed input need to be addressed in future studies.

### 4.3 Limitations

First, the lack of variability, e.g., in the age of acquisition, in our sample did not allow us to examine when the different inflections emerged with various ages of exposure to English. Second, this sample of children with highly educated parents makes extending the findings to lower SES children unsuitable. Third, given the prevalence of Mixed language input, more fine-grained information on the *quantity* and *quality* of English and Mandarin is needed, e.g., using corpus data, to better understand the role of bilingual input in language development. Finally, while the results are consistent with past studies, given the modest sample size, these results ideally need to be replicated in larger samples.

### 4.4 Conclusion

Mandarin-English speaking bilingual 4–6-year-olds demonstrated monolingual acquisition patterns for English inflectional grammar, suggesting that acquiring linguistic structures only in English may follow a monolingual-like pattern. However, given less than 50% English input, a more protracted development course should be expected and persist during early primary schooling. While Mixed language input appears to play a positive role in English acquisition, a better understanding is needed before its practical use becomes clear. The role of English vocabulary and Mixed language input on Mandarin acquisition also requires further investigation.

## Data availability statement

The raw data supporting the conclusions of this article will be made available by the authors, in consultation with the terms of the original ethics approval.

## Ethics statement

The studies involving humans were approved by Macquarie University Human Sciences Ethics Research Committee. The studies were conducted in accordance with the local legislation and institutional requirements. Written informed consent for participation in this study was provided by the participants' legal guardians/next of kin.

## Author contributions

NX: Conceptualization, Data curation, Formal Analysis, Funding acquisition, Investigation, Methodology, Project administration, Resources, Software, Supervision, Validation, Visualization, Writing – original draft, Writing – review & editing. J-HK: Conceptualization, Funding acquisition, Investigation, Methodology, Resources, Supervision, Writing – review & editing.

## Footnotes

^1^In Sydney, all 4-year-olds can begin preschool (provided by childcare or primary schools), 5-year-olds can begin kindergarten (the first year of full-time schooling), and 6-year-olds in year 1.

^2^The activities reported were predominantly eating, playing (at home or the park), watching TV, and doing homework, and 10 children reported participating in extracurricular learning activities, including sports (e.g., swimming) and arts (e.g., piano). The last three activities (watching TV, doing homework, extracurriculars) typically involved English only. The language environments at childcare and schools were reported as English-language only (15) or Mandarin and English (five).

^3^According to Jia et al. (2014), these words were selected from an initial 114 objects and 84 action words which underwent further selection with Mandarin speakers (among others) to create the final 50 objects and 50 action words. These words have high degree of naming agreement by monolingual English and Mandarin speakers.

^4^Examples of the elicitation task are: The progressive, children were shown a picture of a person jogging with the audio “*She likes to jog, she does it every day, so now she is…?*”; For present tense children heard, ‘*…so every day she…*’; For past tense children heard, ‘*… yesterday she…*’; For plurals children first saw a picture with a single animal and heard, e.g., ‘*here is a dog*’ and then saw a picture with two animals and were asked, ‘*What do you see now? These are…*’. Children were trained to provide complete sentences to ensure that inflections were supplied in obligatory contexts, i.e., ‘*she runs*’ and not just run/runs.
